# Bioactive Compounds and Functional Properties of Herbal Preparations of *Cystus creticus* L. Collected From Rhodes Island

**DOI:** 10.3389/fnut.2022.881210

**Published:** 2022-05-23

**Authors:** Andrei Mocan, Ângela Fernandes, Ricardo C. Calhelha, Laura Gavrilaş, Isabel C. F. R. Ferreira, Marija Ivanov, Marina Sokovic, Lillian Barros, Mihai Babotă

**Affiliations:** ^1^Faculty of Pharmacy, “Iuliu Haţieganu” University of Medicine and Pharmacy, Cluj-Napoca, Romania; ^2^Laboratory of Chromatography, Institute of Advanced Horticulture Research of Transylvania, University of Agricultural Sciences and Veterinary Medicine, Cluj-Napoca, Romania; ^3^Centro de Investigação de Montanha (CIMO), Instituto Politécnico de Bragança, Campus de Santa Apolónia, Bragança, Portugal; ^4^Institute for Biological Research “Siniša Stanković”– National Institute of Republic of Serbia, University of Belgrade, Belgrade, Serbia

**Keywords:** bioactive compounds, natural antioxidant, antimicrobial compounds, *Cystus creticus*, herbal preparations

## Abstract

The members of *Cystus* genus are perenial shrubs with a well-established use in traditional medicine. Among these, *C. creticus* is the most popular, herbal preparations obtained from its aerial parts being recognized as antimicrobial, antitumor and anti-inflammatory agents. The present study aimed to evaluate phytochemical profile and bioactive potential of aqueous and hydroethanolic extracts of *C. creticus* aerial parts harvested from two different areas of Rhodes island. LC-DAD-ESI/MS^n^ analysis revealed the presence of myricetin and quercetin glycosides as main compounds, especially in aqueous extracts, being probably responsible for their enhanced antioxidant and antimicrobial potential. On the other side, hydroethanolic preparations exerted a strong anti-inflammatory and anti-biofilm activity. Our findings suggest that the use of solvents with intermediate polarity can assure the best recovery of bioactive compounds from *C. creticus*, increasing the extraction yield for other non-phenolic compounds which can enhance therapeutic potential of the extract through a synergistic action.

## Introduction

In the last decades, there has been a new trend in the preparation and marketing of drugs based on medicinal plants. These preparations, labeled as herbal drugs or phytomedicines, are single plant extracts or fractions, being distinct from the pure chemical entities of molecular drugs. The new plant-derived products are carefully standardized, and their efficacy and safety for a specific application have been demonstrated (1). Thus, plant-based therapeutic agents continue to have scientific, social, and commercial significance (2).

Several *Cistus* species become popular in Mediterranean areas for their use as herbal infusions due to the pleasant flavor and the multiple health-related benefits associated with consumption of this herbal preparation (3). All *Cistus* species are frequently used in many traditional medicines formulas for their antimicrobial (4, 5), antitumor (6), antiviral (7), and anti-inflammatory (8) properties.

At the moment, many manufacturers promote and market *Cistus* herbal infusions (“Cistus tea”) or dietary supplements consisting of this plant material or extracts of it. These products are especially promoted for a high content and a diverse profile of phenolic compounds (including flavonol glycosides or tannins) with an associated strong antioxidant activity or other potential health-promoting effects (i.e., antitumor or anti-inflammatory effects exhibited through reduction of free radicals and oxidative stress markers) (6, 7). Moreover, for the consumer it is of interest which product yields the highest concentrations of bioactive phenolic compounds (9, 10).

*Cistus creticus* L. is a species of shrubby plant in the family Cistaceae (11), known under various local names such as Pink Rock-Rose or Hoary Rock-Rose. Though it usually has pink flowers, of 4.5–5 cm diameter, this species is very variable. It is widely known as a decorative plant, and it is largely distributed in Northern Africa, Western Asia, Caucasus, and Europe (12). Various studies documented the traditional use of this medicinal plant. A recent study by Fakir et al. (13) documented the wild plants collected for medical purposes by local people in the Western Mediterranean Region of Turkey. Among them, *C. creticus* is known not only for its expectorant proprieties, but also as a traditional remedy against constipation. Another study performed by Koçyigit and Özhatay (14) documented the traditional use of this species as a remedy for urethra inflammation, sterility, as stimulant, stomachic, antidiarrhoeic, for snakebites, burns, and wound healing, all this bioactivities being correlated with the presence of volatile fractions (rich in labdane-type compounds) or non-volatile compounds (i.e., phenolic acids, tannins, flavonols) (12, 15).

Previous pharmacological investigations showed that *Cistus* extracts have antimicrobial, antitumor, and anti-inflammatory properties (16, 17). These previous results encouraged us to deepen the studies on these properties, specifically on *C. creticus* collected from two different locations from Rhodes Island (Seven Springs and Valley of Butterflies), by evaluating the antibacterial potential against a broad spectrum of bacteria with relevance for public health, the cytotoxic activity on a large variety of carcinoma cells, the antioxidant capacity, and the anti-inflammatory activity of *Cistus* extracts. The chemical composition was determined using LC-DAD-ESI/MS technique in order to correlate the evaluated biological effects with specific compounds.

## Materials and Methods

### Standards, Reagents, and Other Chemicals

HPLC-grade acetonitrile (99.9%) was obtained from Fisher Scientific (Lisbon, Portugal) and formic acid from Sigma-Aldrich (St. Louis, MO, USA). The phenolic compound standards were from Extrasynthese (Genay, France). Water was treated in a Milli-Q water purification system (TGI Pure Water Systems, Greenville, SC, USA). 2,2-diphenyl-1-picrylhydrazyl (DPPH) was obtained from Alfa Aesar (Ward Hill, MA, USA). Standard trolox (6-hydroxy-2,5,7,8-tetramethylchroman-2-carboxylic acid) was from Sigma (St. Louis, MO, USA). RAW 264.7 cells were purchased from ECACC (“European Collection of Animal Cell Culture”) (Salisbury, UK) and DMEM from Hyclone (Logan, Utah, US). The Griess Reagent System Kit was purchased from Promega (Madison, WI, USA). Dulbecco's modified Eagle's medium (DMEM), hank's balanced salt solution (HBSS), fetal bovine serum (FBS), L-glutamine, trypsin-EDTA, penicillin/streptomycin solution (100 U/mL and 100 mg/mL, respectively) were purchased from Gibco Invitrogen Life Technologies (California, USA). Methanol and all other chemicals were of analytical grade and obtained from local suppliers.

### Plant Material

*C. creticus* ssp. *creticus* aerial parts were collected during flowering period (June 2019) from two distinct areas of Rhodes Island, Greece (Valley of Butterflies–located on Western coast of Rhodes island and Seven Springs–located in Northern part of Rhodes island). Raw material was dried at room temperature during 14 days; the dried plant material was further transported to Romania (packaged in proper conditions), kept away from direct sunlight and stored in paper bags at room temperature in the herbarium of the Department of Pharmaceutical Botany, Iuliu Haţieganu University of Medicine and Pharmacy from Cluj-Napoca (Romania). After authentication procedure (carried out by Dr. Andrei Mocan), samples gathered from the two locations were noted as following: CCV and ICCV are samples (hydroethanolic extract and infusion, respectively) from Valley of Butterflyes; CCI and ICCI are samples (hydroethanolic extract and infusion, respectively) from Seven Springs.

### Extraction Procedure

Hydroethanolic extracts and infusions were prepared from *C. creticus* aerial parts. The hydroethanolic extraction (80% ethanol, 30 mL) was performed by maceration (stirring at 150 rpm), with 1 g of each sample at 21°C during 1 h and then filtered; the residue was re-extracted, using the same methodology. Afterwards, the extracts were evaporated in order to remove the solvent, under reduced pressure (rotary evaporator Büchi R-210, Flawil, Switzerland). For aqueous extracts (infusions), 2 g of plant material was infused with boiling distilled water (200 mL) for 15 min and then filtered. Both extracts were previously frozen before lyophilization (FreeZone 4.5, Labconco, Kansas City, MO, USA), in order to obtain a dry extract (powder). The dry hydroethanolic and infusions extracts were dissolved in ethanol/water (80:20, *v/v*) and water, respectively, to obtain a stock solution of 10 mg/mL for the phenolic compounds characterization; 5 mg/mL for the antioxidant activity; 20 mg/mL in culture medium for the antimicrobial assays; and, finally, 8 mg/mL in water for anti-inflammatory and cytotoxicity tests.

### LC-DAD-ESI/MS^n^ Analysis of Phenolic Compounds

A previous reported method was used to characterize the phenolic profile of the extracts (18). For the identification and quantification of phenolic compounds, a Dionex Ultimate 3000 UPLC (Thermo Scientific, San Jose, CA, USA) system equipped with a diode array detector coupled to an electrospray ionization mass detector (LC-DAD-ESI/MS^n^), a quaternary pump, an auto-sampler (kept at 5°C), a degasser and an automated thermostatted column compartment was employed. A double online detection was preformed using a Diode Array Detector DAD (280, 330 and 370 nm, as preferential wavelengths) coupled with an ESI mass spectrometer working in negative mode (Linear Ion Trap LTQ XL mass spectrometer, Thermo Finnigan, San Jose, CA, USA). Calibration curves were prepared with different available standards; for the identified phenolic compounds for which a commercial standard was not available, the quantification was performed through the calibration curve of the most similar available standard and results were expressed as mg/g of extract (19).

### Antioxidant Activity

The antioxidant activity was measured using four assays, namely 2,2-diphenyl-1-picrylhydrazyl (DPPH) radical-scavenging activity, reducing power, inhibition of β-carotene bleaching and lipid peroxidation inhibition measured by the thiobarbituric acid reactive substances reaction (TBARS) as previously described (20–22). The results were expressed in EC_50_ values (sample concentration providing 50% of antioxidant activity or 0.5 of absorbance in the reducing power assay) and Trolox was used as a positive control.

### Anti-inflammatory Activity Assay

The LPS-induced NO (nitric oxide) production by murine macrophage (RAW 264.7) cell lines was determined as nitrite concentration in the culture medium according to the method previously performed (20). Results were expressed in EC_50_ values (μg/mL), and dexamethasone was used as a positive control.

### Cytotoxicity Assays

The cytotoxicity was evaluated against four human tumor cell lines, MCF-7 (breast carcinoma), NCI-H460 (non-small cell lung cancer), HeLa (cervical carcinoma), HepG2 (hepatocellular carcinoma), and non-tumor porcine liver cell line (PLP2), using the sulphorhodamine assay previously performed by Sobral et al. (20). Results were expressed as GI_50_ (μg/mL), ellipticine being used as a positive control.

### Antibacterial Activity Assays

Antibacterial activity of the extracts was tested against a set of four Gram-positive bacteria, [namely *Bacillus cereus* (clinical isolate), *Micrococcus luteus* (ATCC 10240), *Staphylococcus aureus* (ATCC 6538) and *Listeria monocytogenes* (NCTC 7973)] and four Gram-negative strains [*Escherichia coli* (ATCC 35210), *Pseudomonas aeruginosa* (ATCC 27853), *Salmonella* Typhimurium (ATCC 13311), *Enterobacter cloacae* (clinical isolate)] using p-iodonitrotetrazolium chloride assay. Presence/absence of microbial growth was determined by binocular microscope and the lowest concentration with no bacterial growth was recorded as minimal inhibitory concentration (MICs). Minimal bactericidal concentrations (MBCs) were determined by sub-culturing 2 μL of the medium containing the extract into in 100 μL of medium broth per well. 24 h later the lowest concentration with no visible growth was defined as the MBC. Moreover, we tested the capacity of each extract to inhibit biofilm formation using *P. aeruginosa* PA01 stain. Each test was performed in triplicate, using ampicillin and streptomycin as positive controls (23, 24).

### Statistical Analysis

For each species, three samples were used, and all the assays were carried out in triplicate. Statistical comparisons were performed using an SPSS Statistics v. 23.0 program, and differences were significant at the level of α = 0.05, by using the one-way analysis of variance (ANOVA) followed by Tukey's HSD. When necessary, a Student's *t*-test was used, to determine the significant difference between <3 different samples, with *p* = 0.05. All the data was expressed as means values with the standard deviations (mean ± SD).

## Results and Discussion

### LC-DAD-ESI/MS^n^ Analysis of Phenolic Compounds

The phenolic profile of hydroethanolic extracts and infusions prepared from *C. creticus* aerial parts is presented in Table 1. Based on peak characteristics, retention time, λ_max_, pseudomolecular ion and main fragment ions in MS^2^, seven compounds were tentatively identified. Structurally, all of them are belonging to flavonols subfamily, being recognized as three major types: myricetin derivates, quercetin derivates, and kaempferol derivates.

**Table 1:** Retention time (Rt), wavelengths of maximum absorption in the visible region (λ_max_), mass spectral data, tentative identification and quantification (mg/g of extract) of the phenolic compounds present in *Cistus creticus* herbal preparations (mean ± SD, *n* = 9).

**Peak**	**Rt (min)**	**λ_max(nm)_**	**Molecular ion [M-H]^**−**^(*m/z*)**	**MS^**2**^ (*m/z*)**	**Tentative identification**	**Quantification**			
						**CCI**	**ICCI**	**CCV**	**ICCV**
1	14.88	357	479	317 (100)	Myricetin-3-*O*-glucoside	2.40 ± 0.01c	1.9 ± 0.1d	5.3 ± 0.1a	4.6 ± 0.1b
2	17.45	351	463	317 (100)	Myricetin-*O*-deoxyhexoside	9.8 ± 0.2d	13.9 ± 0.1b	12.4 ± 0.1c	20.69 ± 0.02a
3	18.11	352	463	301 (100)	Quercetin-3-*O*-glucoside	1.17 ± 0.03c	1.72 ± 0.01b	1.8 ± 0.1b	2.6 ± 0.3a
4	21.13	351	433	301 (100)	Quercetin-*O*-pentoside	0.95 ± 0.01a	0.96 ± 0.02a	0.96 ± 0.05a	0.95 ± 0.06a
5	22.32	348	447	447 (100)	Quercetin-*O*-deoxyhexoside	1.91 ± 0.08d	2.47 ± 0.04b	2.22 ± 0.04c	3.08 ± 0.04a
6	27.75	271.320	625	479 (), 317 (10)	Myricetin-3-*O*-glucoside-6″-O- (4-hydroxycinnamoyl) ^*^	0.77 ± 0.01	0.62 ± 0.04	nd	nd
7	32.92	268.311	593	447 (), 285 (100)	Kaempferol-3-O-glucoside-6″-O- (4-hydroxycinnamoyl)	1.73 ± 0.02d	2.7 ± 0.2b	2.24 ± 0.08c	4.3 ± 0.3a
					Total phenolic compounds	18.8 ± 0.4c	24.3 ± 0.1b	24.9 ± 0.2b	36.2 ± 0.7a

* *Nd, not detected; for Myricetin-3-O-glucoside-6″-O- (4-hydroxycinnamoyl) compound, significant differences (p < 0.001) between the two samples were assessed by a Student's t-test. CCI and ICCI- hydroethanolic and infusion extract from Seven Springs, respectively; CCV and ICCV - hydroethanolic and infusion extract from Valley of Butterflyes, respectively. Standard calibration curves used for quantification: Quercetin-3-O-glucoside (y = 34843x - 160173, R^2^ = 0.9998, LOD = 0.21 μg/mL and LOQ = 0.71 μg/mL, peaks 1-6) and Kampherol-3-O-rutinoside (y = 11117x + 30861, R^2^ = 0.9999, LOD = 0.15 μg/mL and LOQ = 0.41 μg/mL, peak 7). The statistical differences in the mean values for the samples CCI, ICCI, CCV, and ICCV were obtained through one-way analysis of variance (ANOVA). In each row, different letters (a, b, c, d) signify significant differences between the samples (p < 0.05)*.

Due to their increased polarity (exerted by the presence of trihydroxylated B ring in flavonol structure) (25), compounds **1** and **2** eluted first in the present chromatographic conditions; they were assigned as myricetin-3-*O*-glucoside [(M–H)^−^ at *m/z* 479] and myricetin-*O*-deoxyhexoside [(M–H)^−^ at *m/z* 479], releasing the same pseudomolecular ion after MS^2^ fragmentation (*m/z* 317), thus compound 1 was positively identified with the commercial standard. Compound **6**, found exclusively in ICCV and CCV samples, presented a fragment attributed to the presence of 6″-*O*-(4-hydroxycinnamoyl) substituent (*m/z* 146), producing two pseudomolecular ions detected in MS^2^ (*m/z* 479 and 317, respectively), being tentatively identified as myricetin-3-*O*-glucoside-6″-*O*-(4-hydroxycinnamoyl).

Quercetin-3-*O*-glucoside [(M–H)^−^ at *m/z* 463] and quercetin-3-*O*-pentoside [(M–H)^−^ at *m/z* 433] were identified based on their common MS^2^ fragment ion at *m/z* 301, due to loss of hexosyl (*m/z* 162) and pentosyl (132 u) moieties, respectively. Their presence as main constituents of different *Cystus* extracts (infusions, methanolic and hydroethanolic preparations) was previously reported by other authors (9, 10, 26, 27), among other glycosylated forms of quercetin. Compound **5**, revealed a similar fragmentation pattern as compounds 3 and 4, being tentatively identified as quercetin-*O*-deoxyhexoside. A similar trend was observed for compound **7**, yielding fragment ions at *m/z* 479 (-146 u; loss of hydroxycinnamoyl residue) and *m/z* 285 (-162 u; loss of hexoside moiety), revealing a similar fragmentation as compound 6, thus being the only kaempferol derivate identified in all the extracts.

The quantitative distribution of the identified compounds varied depending on the extraction method and the sample collection site. Overall, herbal preparations obtained from the raw material collected from Valley of Butterflies (CCV and ICCV) were the richest in phenolic compounds. The effect of the solvent on the extraction yield was also observed, especially for hydroethanolic preparations which exerted high amounts of phenolics in comparison with infusions. Myricetin-*O*-deoxyhexoside was the most abundant compound in all the extracts, followed by myricetin-3-*O*-glucoside. On the other side, distribution of kaempferol glycosylated form was higher in infusions than in hydroethanolic extracts.

### Antioxidant Activity

Many tests have been developed in order to evaluate the antioxidant capacity of foods and biological samples. There is no universal method that can measure the antioxidant capacity of all samples accurately and quantitatively. Thus, it is critical to correlate the radical source and system characteristics to antioxidant reaction mechanisms, with the selection of appropriate assessing antioxidant capacity method/s, as well as the consideration of the end use of the results (28). In this regard, to screen the antioxidant properties of the samples, four *in vitro* assays were performed: DPPH radical scavenging capacity, reducing power (measured by ferricyanide/Prussian blue assay), inhibition of β-carotene bleaching and inhibition of lipid peroxidation in brain cell homogenates by TBARS assay.

The infusion obtained from the aerial parts of *C. creticus* from The Valley of the Butterflies (ICCV) exerted the highest reducing power and radical scavenging properties, whereas the hydroethanolic extract from the same samples exhibited the highest inhibition in terms of lipid peroxidation (significantly lower EC_50_ values; *p* < 0.05) (Table 2).

**Table 2:** Antioxidant, anti-inflammatory and cytotoxic activities of the *Cistus creticus* herbal preparations (mean ± SD, *n* = 9).

	**CCV**	**ICCV**	**CCI**	**ICCI**	
**Antioxidant activity (EC**_**50**_ **μg/mL)**^**a**^					Trolox
Reducing power	132.2 ± 0.8b	117 ± 2c	131.5 ± 0.6b	185 ± 2a	41.7 ± 0.3
DPPH scavenging activity	189 ± 3a	46.1 ± 0.4d	69 ± 2c	85.6 ± 0.8b	41 ± 1
β-carotene/linoleate	14.5 ± 0.3d	92 ± 2a	57.7 ± 0.4c	84 ± 4b	18 ± 1
TBARS	2.2 ± 0.1d	23.5 ± 0.4b	5.5 ± 0.2c	31.3 ± 0.5a	23 ± 1
**Anti-inflammatory activity (EC**_**50**_**;** **μg/mL)**^**b**^					Dexamethasone
Production of nitric oxide (NO) in RAW264.7	147 ± 11b	178 ± 5a	123 ± 8d	136 ± 9c	16 ± 1
**Cytotoxicity to tumor cell lines (GI**_**50**_, **μg/mL)**^**c**^					Ellipticine
HeLa (cervical carcinoma)	59 ± 2b	64 ± 2a	42 ± 2c	59 ± 3b	1.91 ± 0.06
NCI-H460 (non-small cell lung cancer)	61 ± 4b	70 ± 7a	46 ± 2c	61 ± 5b	1.0 ± 0.1
MCF-7 (breast carcinoma)	82 ± 4b	102 ± 11a	58 ± 5c	82 ± 4b	0.91 ± 0.04
HepG2 (hepatocellular carcinoma)	113 ± 11b	127 ± 2a	81 ± 5d	104 ± 11c	1.1 ± 0.2
**Cytotoxicity to non-tumor cell lines (GI**_**50**_, **μg/mL)**^**c**^					Ellipticine
PLP2 (porcine liver primary culture)	300 ± 170a	258 ± 15b	181 ± 4d	228 ± 14c	3.2 ± 0.7

*Trolox, dexamethasone and ellipticine were used as positive controls*.

a *The antioxidant activity was expressed as EC_50_ values corresponding to the extract concentration providing 50% of antioxidant activity or 0.5 of absorbance in the Ferricyanide/Prussian blue assay*.

b *EC_50_ values: correspond to the extract concentration achieving 50% of the inhibition of NO-production*.

c *GI_50_ values: correspond to the sample concentration responsible for 50% inhibition of growth in tumor cells or in a primary culture of liver cells-PLP2*.

*In each row, values for each extract followed by different Latin letters indicate significant differences among the extracts (p < 0.05). CCI and ICCI- hydroethanolic and infusion extract from Seven Springs, respectively; CCV and ICCV- hydroethanolic and infusion extract from Valley of Butterflyes, respectively*.

The best results of antioxidant activity were obtained in the TBARS assay for CCV hydroethanolic extract, with an EC_50_ of 2.2 ± 0.1 μg/mL, followed by CCI hydroethanolic extract (EC_50_ = 5.5 ± 0.2 μg/mL) (Table 2). The same trend was observed for hydroethanolic extracts and infusions from *C. creticus* collected from Seven Springs (CCI and ICCI, respectively). This procedure measures the formation of malondialdehyde (MDA) as the split product from the degradation of an unsaturated fatty acid through an endoperoxide resulting from oxidation of a lipid substrate. According to a previous study (29) the formation of MDA from fatty acids with <3 double bonds (*e.g*., linoleic acid) occurs via the secondary oxidation of primary carbonyl compounds. The MDA react with thiobarbituric acid (TBA) to form a pink pigment (TBARS) that is measured spectrophotometrically at 532 nm (30).

Good results of antioxidant activity were obtained in the β-carotene–linoleate assay, and the values are presented in Table 2. Concerning the obtained results, we can conclude that the trend is the same as in the TBARS assay. CCV hydroethanolic extract exhibited the strongest lipid peroxidation inhibition (EC_50_ = 14.5 ± 0.3 μg/mL), whereas the weakest activity toward lipid peroxidation was obtained for ICCV extract (EC_50_ = 92 ± 2 μg/mL). This is another assay of evaluating the antioxidant activity which consists in monitoring the decolorization of β-carotene using spectrophotometry at 470 nm (31).

The results obtained for lipid peroxidation inhibition can be explained by the fact that the hydroethanolic extract contain a higher number of less polar compounds, and in larger quantities, compared to infusion, thus, exhibiting a more potent lipid peroxidation inhibition than the hydrophilic extract (infusion). Another explanation for this trend can be the fact that the temperature used for infusion might produce a massive degradation of the less polar compounds involved in inhibition of lipid peroxidation (21).

The high values obtained for this assay, especially for hydroethanolic extracts can be ascribed to the intimate antioxidant mechanism which consist in the transformation of Fe^3+^ into Fe^2+^ in the presence of various extracts. The antioxidants present in the plant matrix cause the reduction of Fe^3+^/ferricyanide complex [FeCl_3_/K_3_Fe(CN)_6_] to the ferrous form (Fe^2+^). Therefore, depending on the reducing power of the samples, the color of the test solution changes to various varieties of green or blue (32). The color can be measured spectrophotometrically at 700 nm determining the reducing power of the tested substances using a certain metal responsible for free radicals production and in some cases for antioxidants regeneration. Nonetheless, most of the organic compounds found in plant matrices, are very complex, thus, they cannot exhibit a strong redox potential in order to reduce the Fe^3+^ to Fe^2+^.

Another method used for the evaluation of the antioxidant capacity of plant matrices is DPPH scavenging activity. The most active extract was ICCV, with an EC_50_ of 46.1 ± 0.4 μg/mL. Overall, regarding reducing power assay and DPPH scavenging activity assay, we can conclude that the hydrophilic extracts (infusions) gave better antioxidant activity results than hydroethanolic ones. These results can be at least partially ascribed to a higher quantity of myricetin glycosides found in infusions, compared to extracts, as shown in Table 1.

The results gained from these assays provide simple data that make it possible to classify extracts with respect to their antioxidant potential. Because antioxidant activity does not always correlate with the presence of large quantities of polyphenols, the phenolic content and antioxidant activity data need to be examined, together, when screening plant extracts.

### Anti-inflammatory Activity

Chronic inflammation plays an important role in the development of several pathologic conditions, including malignity, cardiovascular and methabolic diseases, their presence being correlated with high levels of inflammation markers like oxigen (ROS) and nitrogen (NOS) reactive species. Hence, nitric oxide (NO) production can be used as an predictive biochemical assay to evaluate antiinflammatory potential of different bioactive molecules, raw extracts or phytochemicals (20, 33, 34). The effects of the analyzed extracts on the production of NO in RAW264.7 macrophages upon stimulation with LPS, are shown in Table 2.

In terms of IC_50_, LPS-induced NO production was weaker for infusions than hydroethanolic extracts. The most active sample was the hydroethanolic preparation obtained from raw material collected from Seven Springs (123 ± 8 μg/mL), a similar value being observed for ICCI (136 ± 9 μg/mL). This trend can be explained due to the presence of myricetin and quercetin glycosides as main compounds in the extracts, our findings being supported by other previous studies that proven the anti-inflammatory potential of *Cistus x incanus* and *C. laurifolius* extracts rich in this type of compounds (8, 35). Ambrosio et al. (35) studied *in vitro* anti-inflammatory effect of *C. x incanus* ethyl acetate fractionated extract rich in myricetin and quercetin derivates, showing that it can activates Nrf2/HO-1 pathway in LPS-stimulated RAW 264.7 macrophages and decrease mRNA expression of interleukin-6 (IL-6) and cyclooxygenase-2 (COX-2) and the production of prostaglandins E2 (PGE2). Moreover, anti-inflammatory potential of different *Cistus sp*. herbal preparations (aqueous, hydroethanolic or fractionated extracts) was proven through *in vivo* experiments (ex: paw edema model in rats), being also correlated with the presence of phenolic derivates (8, 26, 27, 36). Hence, our findings are in line with previous reports on anti-inflammatory potential of *Cistus sp*. herbal preparations and encourage further investigations on their phenolic-rich fractions in order to establish their deep mechanism of action and obtain standardized products with a proper and well-defined use as adjuvant therapy in inflammatory diseases.

### Cytotoxicity Assays

Cytotoxic activity of the *C. creticus* preparations was evaluated based on their GI_50_ values (concentration that inhibited 50% of the net cell growth), presented in Table 2. Overall, the most active samples were hydroethanolic extracts, the highest inhibitory potential being observed against cervical HeLa (CCI – 42 ± 2 μg/mL) and non-small cell lung NCI-H460 (CCI – 46 ± 2 μg/mL) cell lines. Extracts obtained from raw material collected from Seven Springs exerted the highest cytotoxic effect, this trend being observed for each tested cell line.

Several *Cistus* species were previously evaluated for their cytotoxic properties, showing that these effects are dose-dependent and correlated with the main type of bioactive compounds found in the analyzed matrix. Essential oil isolated from *C. ladanifer* inhibited the growth of MCF7 cell line (37), whereas labdane-type diterpenes from *C. incanus* subsp. *creticus* shown an important cytotoxic effect on NSCLC-N6 cell line (4). On the other side, extracts rich in polar / medium-polar compounds (i.e., flavonoid fraction, medium-polar phenolic fraction, anthocyanin-enriched methanolic extract from flowers and floral buds) isolated from *C. incanus* ssp. *creticus* and *C. salviifolius* showed anticancer activity against human colon cancer cell lines HCT116, human breast cancer cells (MCF-7) and ovarian cancer cells (OVCAR) (17, 38). Our present study is one of the first that reports cytotoxic potential of aqueous preparations obtained from *C. creticus* but, with respect to low evidence-based data on this topic, we aim to use this as a start-point for further investigations that will bring new and clear insights about this bioactive potential of this species.

### Antibacterial Activity

Herbal preparations obtained from different *Cystus* species were traditionally used as remedies for different infectious diseases, further *in vitro* studies proving their ability to inhibit bacterial or fungal growth through different mechanisms (5, 16, 17, 38). Moreover, a prospective, randomized, placebo-controlled clinical study investigated the benefits of a highly polymeric polyphenols-rich extract obtained from *C. incanus* aerial parts in the treatment of symptomatic infection of the upper respiratory tract in humans (7). Hence, we aimed to evaluate antibacterial properties of both aqueous and hydroethanolic extracts obtained from *C. creticus*, the antibacterial effectiveness of this preparations being expressed in terms of MIC (minimum inhibitory concentration) and MBC (minimum bactericidal concentration).

As we can see in Table 3, the lowest values of MIC and MBC were obtained for infusions, emphasizing their superior antibacterial potency in comparison with the hydroethanolic extracts. Both Gram positive and Gram negative stains showed a similar sensitivity after exposure; from the first group, *B. cereus* was the most sensible stain, while *L. monocytogenes* and *E. cloacae* were the most sensible Gram negative bacteria, showing similar MIC and MBC values. Even though, overall, hydroethanolic preparations exerted a weak antibacterial activity, CCI was the most active among analyzed samples (MIC = 0.14 mg/mL and MBC = 0.28 mg/mL for *B. cereus*). As we already observed, this sample showed the lowest total phenolic content after LC analysis (Table 1), so, we can suppose that antibacterial potency of CCI is augmented by the presence of other compounds, probably with non-phenolic or non-polar structure, obtaining a cumulative effect. Likewise, this can be explained by the use of alcohol as co-solvent, increasing the affinity of non-polar compounds for extraction solvent and their extraction yield (21). This hypothesis can be explained based on previous studies that prove antibacterial properties of other *Cistus* species through their non-polar / low-polar constituents, like labdane-type diterpenes (4) and low-polar flavonoid aglycones (38).

**Table 3:** Antibacterial activity and percentage of inhibition of *P. aeruginosa* biofilm after treatment with subinhibitory concentrations of tested *Cistus creticus* herbal preparations.

**Antibacterial activity (mg/mL)**		**CCV**	**ICCV**	**CCI**	**ICCI**	**Ampicillin**	**Streptomycin**
**Gram positive stains**							
*B. cereus*	MIC	0.3	0.19	0.14	0.19	0.17	0.03
	MBC	0.6	0.37	0.28	0.38	0.20	0.07
*M. luteus*	MIC	0.6	0.37	1.2	0.76	0.13	0.07
	MBC	1.2	0.74	2.4	1.5	0.15	0.17
*S. aureus*	MIC	0.3	0.37	1.2	0.38	0.10	0.13
	MBC	0.6	0.74	2.4	0.76	0.20	0.30
*L. monocytogenes*	MIC	0.3	0.37	0.6	0.38	0.20	0.22
	MBC	0.6	0.74	1.2	0.76	0.33	0.40
**Gram negative stains**							
*E. coli*	MIC	1.2	1.6	2.4	1.5	0.18	0.13
	MBC	2.4	3.2	4.8	3	0.27	0.20
*P. aeruginosa*	MIC	0.6	0.37	1.2	0.76	0.40	0.13
	MBC	1.2	0.74	2.4	1.5	0.67	0.23
*S. typhimurium*	MIC	0.6	0.37	0.6	0.76	0.13	0.17
	MBC	1.2	0.74	1.2	1.5	0.20	0.27
*En. cloacae*	MIC	0.3	0.37	0.6	0.38	0.17	0.03
	MBC	0.6	0.74	1.2	0.76	0.20	0.07
***P. aeruginosa*** **biofilm inhibition (%)**							
1/2MIC		79	NE	81.1	79.9	30.9	50.7
1/4MIC		46.7	NE	59.7	20.5	43.5	29
1/8MIC		12	NE	NE	14.6	7.8	11.4

*Ampicillin and streptomycin were used as positive controls; MIC, minimal inhibitory concentration; MBC, minimal bactericidal concentration; NE, no effect*.

*CCI and ICCI- hydroethanolic and infusion extract from Seven Springs, respectively; CCV and ICCV- hydroethanolic and infusion extract from Valley of Butterflyes, respectively*.

Inhibition of *P. aeruginosa* biofilm formation was calculated and expressed as percentage after treatment with sub-inhibitory concentrations (1/2, 1/4 and 1/8 of MIC) of each herbal preparation (see Table 3). Surprisingly, hydroethanolic extracts were the most active; CCI showed the strongest inhibitory potential at 1/2 and 1/4 of MIC (no activity was observed at 1/8 of MIC), being followed by CCV. Infusion obtained from raw material collected from Seven Springs exerted a similar inhibition with CCV at 1/2 of MIC, but weaker at lowest concentrations. Noticeable, ICCV didn't show any inhibitory activity. To the best of our knowledge, our results represent the first report about inhibitory potential of *C. creticus* on *P. aeruginosa* biofilm formation. Corroborated with data from MIC and MBC evaluation, we can conclude that *C. creticus* exerts important antibacterial properties due to the synergistic effect of both polar and non-polar compounds, which can interfere through complex mechanisms with bacterial growth. Moreover, the distribution of these compounds in herbal preparations obtained from this species is dependent by the extraction solvent used, intermediate polarity of the solvent ensuring the recovery of a broad range of compounds and an augmented antibacterial effect.

## Conclusion

*C. creticus* aqueous and hydroethanolic preparations represent an important source of bioactive compounds, most of them belonging to big polyphenols family. Myricetin and quercetin derivates are the main constituents, their different distribution in the analyzed samples being a consequence of harvesting place and the type of solvent used in extractive process. High amounts of phenolics were quantified in hydroethanolic extracts, found as the most active as antioxidant, cytotoxic and anti-inflammatory agents. The screening for antibacterial activity proven that infusions can act as bactericidal agents, while their anti-biofilm activity was weaker than for hydroethanolic extracts. Overall, our findings suggest that the use of solvents with intermediate polarity can assure the best recovery of main compounds from *C. creticus*, obtaining herbal preparations with improved bioactive potential. Additionally, this type of solvent can increase the extraction yield for other non-phenolic compounds which can enhance therapeutic potential of the extract through a synergistic action.

## Data Availability Statement

The raw data supporting the conclusions of this article will be made available by the authors, without undue reservation.

## Author Contributions

AM, ÂF, LG, MI, and MB were involved in data processing and manuscript preparing. AM, IF, MS, and LB were involved in research funding. AM, ÂF, RC, MI, and MB were involved in experimental design. All authors listed have made a substantial, direct, and intellectual contribution to the work and approved it for publication. All authors participated in data analysis, correcting the draft and approving the final version of the draft.

## Funding

This work was supported by a grant of the Romanian Ministry of Education and Research, CNCS–UEFISCDI, project number PN-III-P2-2.1-PED-2019-5360. This research was funded by the Serbian Ministry of Education, Science and Technological Development (Contract No. 451–03–9/2021–14/200007).

## Conflict of Interest

The authors declare that the research was conducted in the absence of any commercial or financial relationships that could be construed as a potential conflict of interest.

## Publisher's Note

All claims expressed in this article are solely those of the authors and do not necessarily represent those of their affiliated organizations, or those of the publisher, the editors and the reviewers. Any product that may be evaluated in this article, or claim that may be made by its manufacturer, is not guaranteed or endorsed by the publisher.
